# Obstetric Medicine: the protocol for a prospective three-dimensional cohort study to assess maternity care for women with pre-existing conditions (ForMaT)

**DOI:** 10.3389/fmed.2023.1258716

**Published:** 2024-01-11

**Authors:** Elena Jost, Philipp Kosian, Gregory Gordon Greiner, Andrea Icks, Marie-Therese Schmitz, Matthias Schmid, Waltraut M. Merz

**Affiliations:** ^1^Department of Obstetrics and Prenatal Medicine, University Hospital Bonn, Bonn, Germany; ^2^Institute of Health Services Research and Health Economics, Faculty of Medicine, Center for Health and Society, Heinrich Heine University Düsseldorf and University Hospital, Düsseldorf, Germany; ^3^Institute of Health Services Research and Health Economics, German Diabetes Center (DDZ), Leibniz Institute for Diabetes Research Germany, Düsseldorf, Germany; ^4^German Center for Diabetes Research (DZD), München-Neuherberg, Germany; ^5^Department of Medical Biometry, Informatics and Epidemiology, University Hospital Bonn, Bonn, Germany

**Keywords:** Obstetric Medicine, severe maternal morbidity, rare disease, high-risk pregnancy, maternal pre-existing condition, maternity care, health care costs, pregnancy experience

## Abstract

**Background:**

Pregnancies in women with pre-existing medical conditions are on the rise. These pregnancies are characterized by an increased rate of maternal and perinatal complications, which can result in higher health care expenditures and altered pregnancy experiences. The purpose of this study is to integrally analyze maternity care for women with pre-existing conditions in the framework of a risk-adapted, interdisciplinary care by recording three substantial parts of maternity care: (1) maternal and perinatal outcome; (2) hospital costs and reimbursements covering the period from preconception counseling or initial antenatal visit to discharge after birth; and (3) women’s experience of reproductive choice and becoming a mother in the presence of a pre-existing condition.

**Methods:**

In this observational, prospective, longitudinal, and monocentric cohort study, we aim to include a total of 1,500 women over a recruitment period of 15 months. Women registering for care at the Department of Obstetrics and Prenatal Medicine, University Hospital Bonn, Germany, are allocated to three groups based on their health and risk status: women with pre-existing conditions, as well as healthy women with obstetric risk factor and healthy women with a low-risk pregnancy. Participants are observed from time of initial consultation until discharge after birth. Analysis focuses on (1) maternal and perinatal outcome, especially rate of severe maternal and neonatal morbidity; (2) costs and reimbursements; and (3) surveys to capture of women’s experience and health-related quality of life during the time of reproductive choice, pregnancy, and childbirth in the presence of pre-existing medical conditions.

**Discussion:**

With its complex three-dimensional design, the ForMaT-Trial is aiming to provide a comprehensive analysis of pregnancy and childbirth in women with pre-existing conditions. The results may serve as a basis for counseling and care of these women. By analyzing costs of specialized care, data for discussing reimbursement are generated. Lastly, our results may increase awareness for the perception of reproductive choice, pregnancy and motherhood in this continuously rising population.

**Clinical trial registration**: German Clinical Trials Register, DRKS00030061, October 28, 2022.

## 1 Introduction

### 1.1 Background and rationale

The number of pregnancies in women with pre-existing medical conditions has increased over the last decades (1–3). A Danish registry study of more than 1.3 million births found an increase in the prevalence of maternal chronic conditions over a period of 25 years from 3.7% in 1989 to 15.7% in 2013 (1).

Three main factors contributing to this ongoing trend can be identified. First, the shift to delay childbearing resulting in advanced maternal age is associated with a higher prevalence of chronic conditions, such as cardiovascular diseases, obesity, or diabetes (4, 5). Second, advances in the treatment of severe childhood conditions result in higher life expectancy of affected women, who enter adulthood and may consider pursuing pregnancy. Improved survival rates of childhood, adolescent, and young adult’s cancers (6) or of newborns with congenital heart disease (7–9) may serve as examples. Lastly, progress in assisted reproductive technologies allows pregnancies in women with reduced fertility due to their advanced maternal age or pre-existing condition (10).

Pregnancies in women with pre-existing conditions represent high-risk pregnancies and bear the risk of high-risk deliveries (11, 12). Maternal complications during pregnancy and childbirth, whether they occur because of the underlying chronic condition, obstetric risk factors, or both, may result in severe maternal morbidity (SMM) (12, 13). SMM is defined as a spectrum of complications during pregnancy, childbirth, and postpartum, which results in significant short-or long-term health issues for women including maternal death (14–16). The reasons for higher rates of adverse pregnancy outcomes in women with pre-existing conditions arise from the interactions of chronic disease and pregnancy itself. These may vary upon the underlying condition: While cardiac or renal conditions tend to deteriorate during pregnancy (17, 18), women affected by rheumatic disease or multiple sclerosis often enjoy a relapse-free time during pregnancy but are at risk of suffering from flares in the postpartum period (19–21). However, SMM not only affects maternal health but may also lead to perinatal morbidity and mortality. The spectrum of perinatal consequences includes prematurity, neonatal intensive care unit (NICU) admission, low birthweight, low APGAR-score, or perinatal death (11, 22, 23). Additionally, women with chronic health conditions may require long-term medication, but inadvertent intake of teratogenic or fetotoxic substances may negatively impact the intrauterine development. However, continuation of disease-specific medication is often indispensable for the women’s health (24).

For the above-mentioned reasons pregnancies and deliveries in women with pre-existing conditions are known to entail higher health care expenditures. Risk-adapted antenatal care may cause additional costs on the one hand, but expenses may be saved because of averted complications of childbirth (25). In Germany, reimbursement of antenatal care performed by hospital outpatient departments is regulated by the German Federal Joint Committee (26). However, costs for the time-and intervention-intensified risk-adapted maternity care in women with pre-existing conditions are likely to exceed this reimbursed sum.

A further aspect related to this topic involves the experience of becoming a mother in the presence of a pre-existing condition. The perception of pursuing pregnancy and becoming a mother has been investigated for specific conditions and in small sample sizes (27–30).

The management of pregnant women with pre-existing conditions requires knowledge of the interactions between chronic condition and pregnancy. The German maternity guidelines provide detailed instructions on the obstetric and fetal surveillance but lack specific instructions for counseling and treatment of women with pre-existing conditions (31). Furthermore, the understanding and management of chronic conditions during pregnancy, childbirth and postpartum period is being taught in the growing field of Obstetric Medicine as a subspeciality of internal medicine in only few countries, such as Canada, Australia and the United States of America (32). In Germany, only few university hospitals hold outpatient consultations for specific diseases in pregnancy but specialized sections covering all medical conditions are not yet established. At the Department of Obstetrics and Prenatal Medicine, University Hospital Bonn, Germany, women with pre-existing conditions are cared for within the frame of a specialized consultation. This includes preconception counseling and care during pregnancy, childbirth, and the puerperium within an interdisciplinary framework.

### 1.2 Study aim

This study is designed to describe risk-adapted maternity care for women with pre-existing conditions on a medical, economic, and psychological level. By assessing rate of SMM among this special population data will be generated which may serve as a basis for recommendations of the care of women with pre-existing conditions during reproductive choice, pregnancy and postpartum period. Consequently, establishing a framework for the care of affected women is aim of this study. Furthermore, by capturing and analyzing costs and reimbursement of specialized care, data will be generated which may serve as basis for discussion with the relevant bodies. Lastly, the special perceptions and needs of affected women will be elucidated to provide care givers with evidence and to increase awareness for this steadily increasing group of patients.

### 1.3 Objectives

The objectives of this three-dimensional study include the assessment of:

1. maternal and perinatal outcome with a special focus on the SMM rate;
2. services and associated costs covering the period from preconception counseling or initial antenatal visit to discharge after birth;
3. women’s experience of reproductive choice and becoming a mother in the presence of a pre-existing condition.

## 2 Methods

### 2.1 Trial design and study status

This study is designed as an observational, prospective, longitudinal single-center cohort study. Recruiting and data collection started in November 2022 and is scheduled to last until January 2024. Observation of participants covers the period from preconception counseling or initial consultation in pregnancy to hospital discharge after birth. Assignment to study and comparative groups is applied during recruiting.

This protocol is structured in accordance with the guidelines of Standard Protocol Items: Recommendations for Interventional Trials (SPIRIT)-Patient-Reported Outcomes (PRO)-Extension (33–35), although it is not considered an interventional trial. This extended version addresses the importance of patient-centered care by implementing recommendations to correctly document PRO in clinical trial protocols. In addition to this specific protocol writing guideline, we also applied aspects of the Strengthening the Reporting of Observational Studies in Epidemiology (STROBE) guidelines as this study is designed as an observational cohort study (36, 37).

Note on language usage: in this work we use the terms “woman/women” to refer to individuals with a uterus and the biological capacity to become pregnant.

### 2.2 Study and health care setting

Study center is the Department of Obstetrics and Prenatal Medicine, University Hospital Bonn, Germany, which serves as a regional referral center and has a unique standing in Obstetric Medicine. The specialized outpatient department “Obstetric Medicine” cares for an increasing number of women with pre-existing medical conditions, which shapes the obstetric patient population and medical education at the department. It routinely interacts with related medical subspecialities and closely cooperates with the Center for Rare Diseases of the University Hospital Bonn.

All women presenting at the study center receive the same risk-adapted obstetric care during pregnancy and childbirth, irrespective of their participation in the study. While women with pre-existing conditions may require more frequent follow-up, interdisciplinary management, case conferences, and additional diagnostic investigations compared to healthy women with obstetric complications, healthy, “low-risk” women register for birth via a midwife appointment. This type of risk-adapted care is the standard of care at the study center and therefore is not considered a classical study intervention.

### 2.3 Eligibility criteria

Women in all stages of pregnancy and women seeking preconception counseling who present at the study center can be included in this study if they meet the inclusion criteria and provide informed written consent. All participating women must be capable to fully understand the study requirements and intentions. The study group (S) is compared to two additional groups (K1 and K2). The inclusion criteria vary as follows.

#### 2.3.1 Study group S

Women with pre-existing medical conditions, presenting either pregnant or for preconception counseling, are invited to join the study. For our study, we defined pre-existing condition as the presence of chronic diseases (e.g., multiple sclerosis) or of sequelae of a preceding health incident (e.g., history of pulmonary embolism). A list of included conditions can be found in Supplementary Table 1. It is important to emphasize the fact that only severe conditions, which may have an impact on the course of pregnancy and/or delivery and/or on women’s perception of pregnancy are included. Therefore, pre-existing conditions which appear to be irrelevant for pregnancy outcome and women’s perception are explicitly excluded. For the latter, in Supplementary Table 2, we provide a list of conditions considered to be irrelevant for our investigation, meaning that women exclusively fulfilling these diagnoses are not included in the study (e.g., essential hypertension without medication before pregnancy). Both selection processes were undertaken by consensus meetings of the research team. Uncertainties regarding inclusion or exclusion of rare or exceptional cases are resolved in a consensus meeting with two Obstetric Medicine experts on a case-by-case decision (e.g., sarcoidosis without functional impairment).

Women in study group S are required to understand and complete the questionnaires in German language. If a woman is willing to participate in the study but not capable to complete the questionnaires due to language barriers, recruitment is possible for analysis of medical outcome and economic evaluation only.

#### 2.3.2 Additional group K1

Healthy pregnant women whose pregnancies are considered at-risk due to their obstetric history or findings during the current pregnancy (e.g., gestational hypertensive disorders, gestational diabetes, and previous cesarean section) are enrolled. No maternal pre-existing condition, except for uncomplicated, substituted hypothyroidism, must be present. A list of all pre-defined obstetric conditions and complications is provided in Supplementary Table 3.

#### 2.3.3 Additional group K2

Healthy pregnant women with a singleton fetus in cephalic presentation who register for birth at term after an uneventful course of pregnancy—i.e., low-risk women expecting an uncomplicated vaginal delivery—are invited for study participation as a second comparative group. No maternal pre-existing condition or obstetric complication, except for substituted hypothyroidism, must be present.

#### 2.3.4 Exclusion criteria for all groups

Women presenting with higher-order multiple pregnancies (more than twin pregnancy) and women who are not capable to understand the study intention are not enrolled. Furthermore, healthy women referred for care at our institution because of fetal anomalies (e.g., aneuploidies, malformations) are not included in the study.

### 2.4 Recruitment

Recruitment started on November 1, 2022 and is scheduled for a period of 15 months. All women presenting at the study center as outpatients or are admitted or referred for inpatient care are screened for eligibility by trained members of the study team. For S and K1 cases, a specialist in obstetrics with expertise in Obstetric Medicine confirms diagnosis and obstetric findings before recruitment. For K2, a midwife performs recruitment after assessment during booking. Additionally, all inpatient admissions are screened on an everyday basis for eligibility. S and K1 also include pregnant women who are admitted to the inpatient department or transferred from other hospitals due to maternal or obstetric complications, and who have not previously presented to the study center for co-care. In selected cases where maternal complications of the pre-existing condition require immediate delivery, postpartum recruitment is possible (e.g., admission with status epilepticus and suspected placental abruption). If a woman is eligible for enrollment, she is provided a printed study information, personal explanation of the study content by a member of the study team and gives informed written consent.

### 2.5 Assignment and stratification

**Figure 1:**
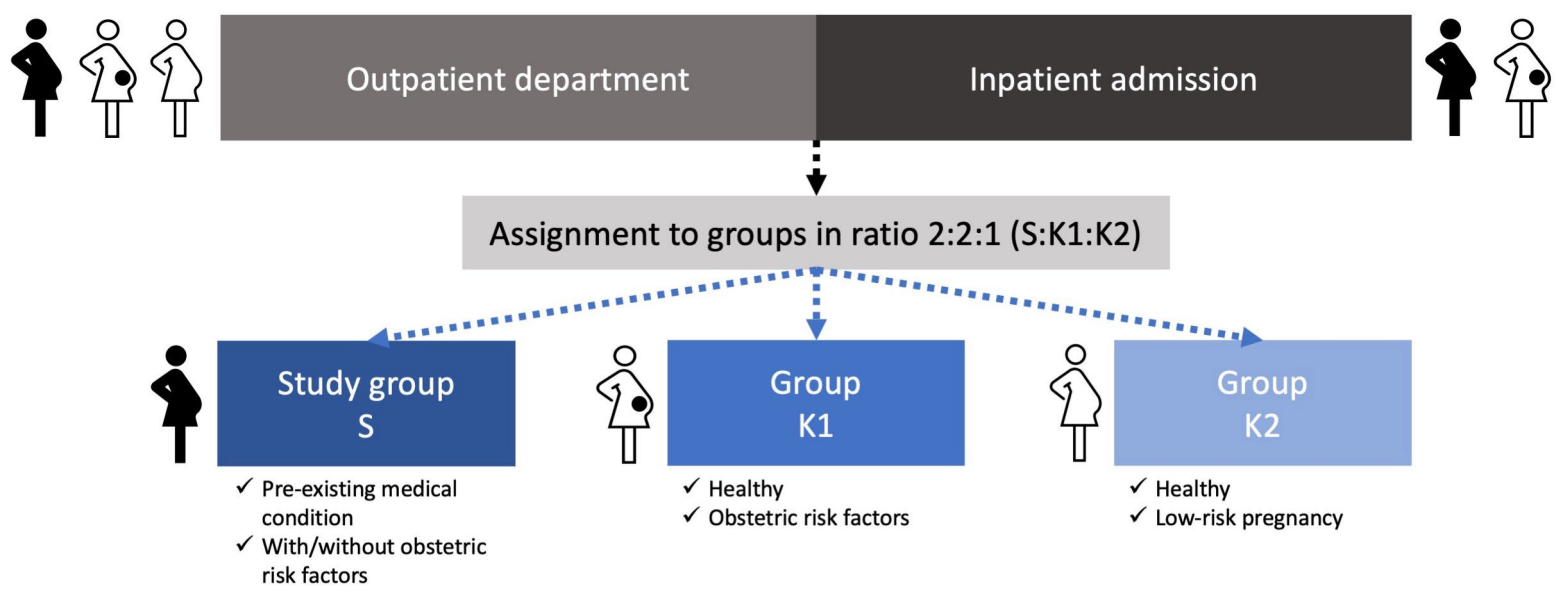
Overview of the recruitment and assignment process for study group S and groups K1 and K2.

Assignment to the groups and stratification is applied during recruitment by the enrolling investigator in a ratio of 2:2:1 (S:K1:K2). This ratio arises from the expected homogeneity of K2, a resource-efficient project planning and the already well-investigated population represented by K2 in existing literature. If uncertainties regarding assignment to the correct group arise, consensus meetings are held to resolve the issue. In Figure 1, we provide an overview of the recruitment and assignment process.

Additionally, we stratified the recruited women as follows:

1. parity (nulliparous vs. parous);
2. singleton vs. twin pregnancy; and
3. outpatient vs. inpatient recruitment.

The rationale for stratification is the difference in the risk profiles of nulliparous vs. parous women and singleton vs. twin pregnancies. The same applies for women requiring inpatient care vs. outpatient consultation.

For every pregnant woman recruited in S, the subsequent pregnant woman who presents at the study center and meets the inclusion criteria for K1 and K2 is invited to participate. To ensure a complete and continuous stratification process analogous lists are compiled, which are only accessible to the members of the research team and are carefully handled in accordance with data protection regulations.

### 2.6 Participant timeline

**Figure 2:**
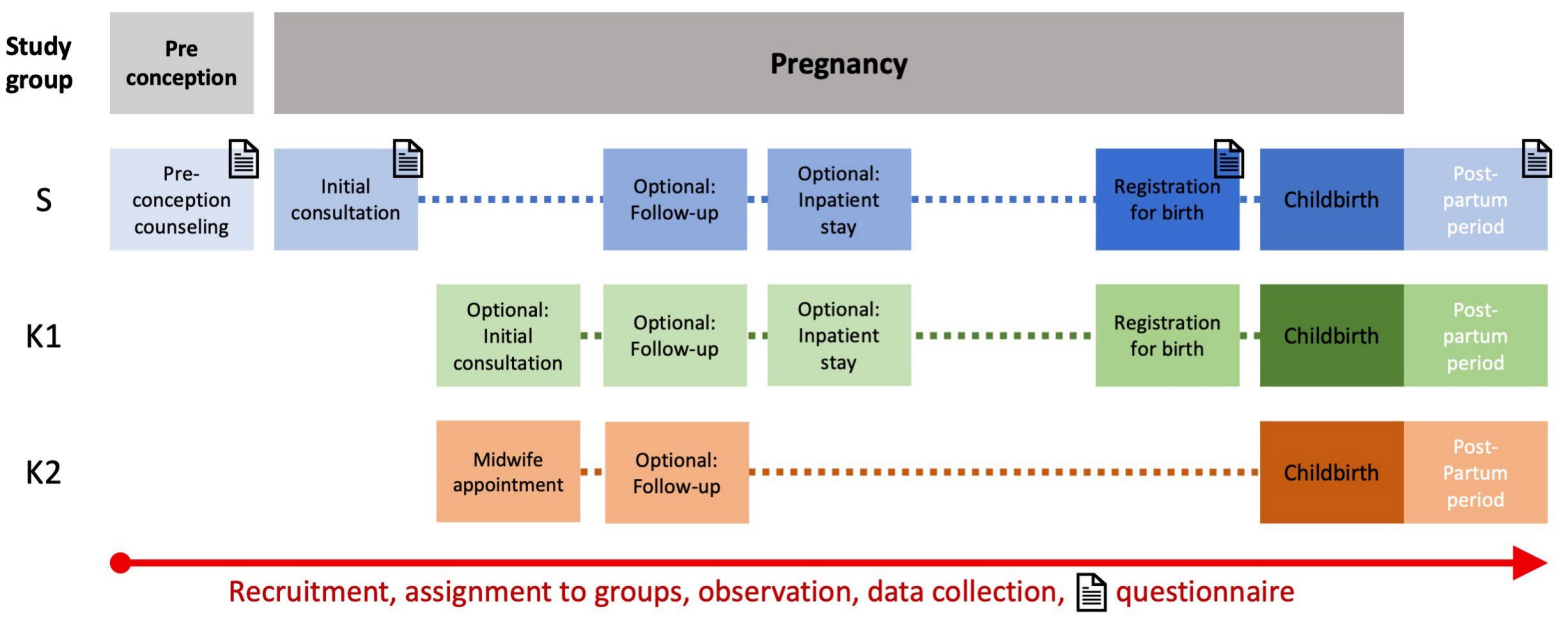
Participant timeline for all three groups from initial consultation (during pregnancy or preconception) to discharge after inpatient stay for childbirth. Timepoints for questionnaire assessment in the study group are marked with letter icons.

An overview of the participant timeline is visualized in Figure 2. Observation in S can start with preconception counseling or at any stage of pregnancy. Observation in K1 normally starts with the registration for birth but may also occur at an earlier stage of pregnancy due to obstetric complications. Observation in K2 starts with recruitment at booking. For all study participants, observation ends with discharge after delivery. Women recruited at preconception counseling who do not become pregnant during the recruitment period are excluded from analysis and studied separately.

### 2.7 Outcome measures

The three-dimensional design of the ForMaT-Trial indicates three different outcome measures as follows:

1. The primary medical outcome is SMM. For its assessment, a list with previously defined indicators can be found in Table 1. It was adapted from the Centers for Disease Control and Prevention (CDC) (15) after a thorough literature review. Maternal secondary outcome measures consist of complications of pre-existing disease or obstetric complications, mode of delivery, type of anesthesia, length of hospital stay for delivery, and need for transfusion >4 units of packed red blood cells (PRBC). Perinatal secondary outcome measures are late miscarriage (>14 weeks of gestation), intrauterine fetal death, gestational age at birth, birthweight with percentile, APGAR-scores, arterial umbilical cord pH, severe neonatal complications including transfer to NICU, length of stay in NICU, and death.
2. In the second part of our study, an analysis of associated costs and reimbursement for study participants is undertaken. This includes the evaluation of billing and cost coverage of the in-and outpatient health sector and further associated costs.
3. The third trial part captures the experience of pregnancy planning, pregnancy, and childbirth, as well as health-related quality of life in women with pre-existing disorders, which is assessed by questionnaires at predetermined time points (Figure 2). The questionnaires are a combination of pre-validated instruments and specifically designed questions. As validated PRO-instruments, the German version of the EQ-5D-5L™ is incorporated in each questionnaire (38) and the German version of the BSS-R (Birth Satisfaction Scale-Revised) is included in the postpartum questionnaire (39, 40). Table 2 provides an overview of item count, domains that are interrogated, and content of all four questionnaires. Questionnaires are only filled out by women in study group S. Timepoints are as follows (Figure 2): during the initial antenatal consultation at the study center; during the appointment for birth planning; and in the postpartum period before discharge from the maternity department. If the initial visit to the study center occurs after 34 weeks of gestation only one antenatal questionnaire is filled out. Recruited women seeking preconception counseling are also asked to fill out a questionnaire after the appointment.

**Table 1:** Indicators of SMM, adapted from the definition of the Centers for Disease Control and Prevention (CDC) (15).

Indicators for severe maternal morbidity (SMM)	ICD-10 short/OPS-Codes
Acute renal failure	N17.-, O90.4
Cardiac arrest/ventricular fibrillation	I46.-; I49.0-
Heart failure/arrest during surgery or procedure	I97.12-; I97.13-; I97.710; I97.711
Shock	O75.1; R57.-; R65.2!; T78.2; T88.2; T88.6; T81.1
Sepsis	O85; T80.2; T81.4, R65.-!, A40.-; A41.-; A32.7
Disseminated intravascular coagulation	D65; D68.8; D68.9; O72.3
Amniotic fluid embolism	O88.1-
Air and thrombotic embolism	I26.-; O88.0-; O88.2-; O88.3-; O88.8-
Puerperal cerebrovascular disorders	I60-I69; O87.1; O87.3
Severe anesthesia complications	O74.- O89.-
Pulmonary edema/acute heart failure	J81.; I50.-
Adult respiratory distress syndrome	J80.-
Acute myocardial infarction	I21.-, I22.-
Eclampsia	O15.-
Sickle cell disease with crisis	D57.0-
Aneurysm	I71.-; 72.-
Ventilation	OPS 8–713
Temporary tracheostomy	OPS 5–311
Conversion of cardiac rhythm	OPS 8–64
Transfusion of blood products (≥4 PRBC or ≥ 2 red PRBC + ≥2 fresh frozen plasma)	OPS 8–80,8–81
Hysterectomy	OPS 5–75
Maternal death	O97.-

**Table 2:** Item count and domains of the four questionnaires at the pre-defined time points (see Figure 2).

	Questionnaire 1	Questionnaire 2	Questionnaire 3	Questionnaire 4
Time points	Preconception counseling	Initial consultation	Birth planning	Postpartum period
Number of items	38	53	56	48
Domains	Inner conflicts Barriers Motivation Social support system Sources and quality of information	In addition to domains of Questionnaire 1: Perception of pregnancy Course of pre-existing condition	In addition to domains of Questionnaire 1: Perception of pregnancy Course of pre-existing condition Issues of childbirth	In addition to domains of Questionnaire 1: Breastfeeding Preparedness for postpartum period
EQ-5D-5L™, 6 items	✓	✓	✓	✓
BSS-R, 10 items				✓

Domains specifically refer to the pre-existing conditions.

Further items were established in a multistep process. An initial collection of main topics was developed via literature research, discussed in a consensus meeting with the team of the Center for Rare Diseases of the University Hospital Bonn, and transferred into a catalog of questions by the interprofessional study team. As an important next PRO-measure step, the catalog of questions was sent to 15 national umbrella organizations of patient advocacy groups, such as adults with congenital heart disease, inflammatory bowel disease, or familial adenomatous polyposis. The feedback and comments of affected persons were included in the catalog and an expert in statistics ultimately proofread the questionnaires. Thereafter, a pre-test was performed by at least two affected women, midwifes, medical students, and physicians.

### 2.8 Sample size

Sample size estimation was based on the primary medical outcome SMM. For the estimation, both additional groups (K1, K2) were pooled which results in a matching ratio of 2 women (S) vs. 3 women (K1 + K2). Following the multiply matched study design, a score test established on a conditional logistic regression model was used (41). The test assesses the effect of a binary variable (= with/without pre-existing medical condition) on the outcome with a variable number of cases and controls in a matched set. Assuming a SMM rate of 50 per 1,000 women in S and a SMM rate of 23/1000 in K1 + K2 257 matched sets of size 5 (2:3) are required to provide 90% power at a significance level of 5%. Considering a drop-out rate of about 15%, 1,500 women in 300 matched sets of size 5 have to be recruited.

### 2.9 Data collection and management

Trial data consist of three categories: medical outcome data, economic data, and questionnaires. Medical outcome is collected via database query of the hospital information system ORBIS (Dedalus Healthcare, Bonn, Germany), the ultrasound software ViewPoint™ (Version 6, GE HealthCare, Solingen, Germany), and the pediatric software Neodat (Version 5, Paedsoft, Tuebingen, Germany). Economic data are collected via database query of the information system SAP (SAP R/3 ECC 6.0, IS-H Release 617 Package, Walldorf, Germany). All questionnaires are completed online with the assistance of a study team member. Data management and monitoring is performed by the Clinical Support Study Center of the University Hospital Bonn, Bonn, Germany. All relevant trial data are entered in a specifically build REDCap© database (Version 12.0.27, Nashville, Tennessee, United States).

### 2.10 Statistical analysis

All medical parameters will be summarized using descriptive statistics. The primary medical outcome will be analyzed with a conditional logistic regression model to assess the effects of pre-existing medical conditions on the SMM rate. Odds ratios obtained from this model with corresponding 95% confidence intervals will be reported. Further secondary medical maternal and perinatal parameters will be compared between the study and additional groups. Additional analyses of medical parameters will be considered in subgroups given by different pre-existing conditions in the study group and are exploratory in nature. The main analysis will be performed on complete cases and the significance level will be set to 0.05. A sensitivity analysis will include the use of multiple imputation strategies to address possible missing observations.

For analysis of costs, a health economic evaluation to determine the costs associated with the interventions and services in the different groups will be conducted. This includes, for example, consultation time, laboratory tests, medical procedures, hospitalization, or medication. The resource use of implemented care of all the three groups will be captured, both for inpatient and outpatient setting. Thereafter, this resource use will be multiplied times unit prices to derive respective costs from a provider (in-and outpatient healthcare provider) and healthcare system perspective (statutory health insurance).

The items of the questionnaires will be summarized descriptively at each time point. Health-related quality of life will be analyzed as pre-specified in the manual of the EQ-5D-5L™ tariff and compared over time (42). The birth experience assessed by the German version of the BSS-R will be reported via the total score and the three subscales (stress during labor, personal attributes, and quality of care provision) (39).

## 3 Discussion

Pregnancies in women with pre-existing conditions are constantly on the rise. In a German survey from 2019/2020, one third (33.8%) of 18–29 year old women reported the presence of at least one long-term health condition, compared to 18.9% women in 2010, representing a 78.8% increase within the last 10 years (4). For the United Kingdom, Lee et al. (2) stated that 20–44% of women entered pregnancy with pre-existing multimorbidity, defined as the coexistence of two or more long-term conditions.

This study targets to integrally analyze risk-adapted obstetric care for women with pre-existing conditions by evaluating medical outcome of mother and newborn, economic impact of specialized care, and women’s experience of pregnancy and childbirth. The intention is to create a holistic view on the current situation of obstetric care for women with pre-existing conditions by conducting a three-dimensional study. Overall strength of this study is the prospective longitudinal design. This design enables the investigators to fully display the complications of maternal pre-existing conditions during pregnancy and assess SMM compared to retrospective trials which are mainly based on data collected for billing purpose (43).

To assess medical outcome, SMM was defined as the primary outcome parameter. To date, there is no standard definition of SMM. The CDC established a list of 21 indicators and ICD-10 codes for SMM, which are commonly applied (15, 16, 43). The original list of 25 indicators with ICD-9 codes was published by Callaghan et al. (44) in 2012 and revised by the CDC. As we use the adapted CDC-list in our study, comparison to the existing literature is facilitated. Furthermore, the presented study owns several important advantages. The recruitment of two additional groups and the application of three stratification criteria allows a sophisticated assessment according to the women’s risk profiles. Lastly, the inclusion of pre-existing disorders from all medical specialties and the strict exclusion of non-relevant pre-existing disorders is what separates this study from other clinical trials. With our study design we are achieving a high quality of evidence-based data.

Pregnancies and deliveries in women with pre-existing conditions are known to imply higher health care costs and we expect to confirm and extend the available evidence presented by Admon et al. (12). This research group analyzed a representative sample of more than 1.5 million inpatients in the United States of America and found a positive association between the number of pre-existing conditions and delivery-associated costs (12).

The record of economic data in this trial will provide a presentation of services and associated costs in maternity care for women with pre-existing conditions across the health sectors in Germany. Through the systematic capture of consultation times and interventions, description of healthcare costs of maternity care in different risk groups is possible. Germany’s healthcare system has some specific features originating from its Bismarckian statutory health insurance system. The health care system in general is ruled by a self-administration body, the Federal Joint Committee (G-BA) and thus the costs are controlled by this body, which determines the benefits catalog and ensures quality and cost-effectiveness by law. Healthcare providers are mainly paid on the basis of fees negotiated between the National Association of Statutory Health Insurance Physicians (KBV) and National Association of Statutory Health Insurance Funds (GKV SV). This definitely has an impact on access to care, innovation, patient outcomes and the behavior of healthcare providers (45).

Data on women’s experience of planning and becoming a mother in the presence of a pre-existing condition derive from small sample sizes but revealed to be of enormous importance (27–30). Iezzoni et al. (46) analyzed peer recommendations for pregnant women with disabilities summarizing five main topics, including the lack of information about the interaction between pregnancy and health condition, access to health care, the feasibility of becoming pregnant and giving birth in the presence of a disability, building support systems, and managing fears. Additionally, a recent review by Hammarberg et al. (47) stated the importance of adequate communication between affected women and healthcare professionals, which was found to be consistent in all studies, including the significance of personalized preconception health information.

For the assessment of women’s perception in our study, questionnaires were developed as a combination of pre-validated PRO-instruments and newly designed items. So far, no suitable questionnaire for the universal assessment of pregnancies with medical conditions has been published. Our questionnaire allows an analysis regardless of the underlying disease. To integrate PRO-instruments, we included the German version of the EQ-5D-5L™ to objectively assess the current health-related quality of life at different timepoints (38, 48). The EQ-5D-5L™ was selected for its simplicity, its recommended application for economic evaluation and its significance as one of the most applied generic health-related quality of life measures worldwide. This instrument is relevant to both healthy and ill subjects, can easily be self-completed, and is intended to be an ‘add-on’ instrument to other questionnaires (49). Furthermore, the postpartum questionnaire in this study includes the BSS-R which was selected as an internationally accepted, validated, robust tool to assess birth satisfaction (39, 40).

There are some limitations to this study. One limiting aspect is the monocentric design, which only allows description of the situation in the specific risk-adapted care at the study center. The most important limitation to this study is its relatively short observation period. It is well known that maternal health and complications during pregnancy and childbirth can imply lifelong sequelae to a woman’s and her offspring’s physical and mental health (50, 51). Specifically, it is known that a proportion of cases with SMM occurs in the weeks postpartum. Since the current study is limited to observation until hospital discharge after delivery, assessment of SMM and medical outcome in childbearing women with pre-existing conditions may be underestimated (16, 44, 52). To address this limitation, we aim to extend our study to include the postpartum period and the first year after birth.

In conclusion, the purpose of this study is to assess the requirements of care for women planning and entering pregnancy with pre-existing conditions. The results of the Format-Trial may lead to improvement of care and treatment for this specific obstetric population, provide basis for a reasonable accounting and reimbursement system and sharpen the awareness for the special needs in this continuously growing number of women.

## Ethics statement

The studies involving humans were approved by Ethics Committee of the Faculty of Medicine, Rheinische Friedrich-Wilhelms University, Bonn. The studies were conducted in accordance with the local legislation and institutional requirements. The participants provided their written informed consent to participate in this study.

## Author contributions

EJ: Conceptualization, Investigation, Validation, Visualization, Writing – original draft. PK: Conceptualization, Investigation, Supervision, Validation, Visualization, Writing – review & editing. GG: Formal analysis, Writing – review & editing. AI: Formal analysis, Writing – review & editing. M-TS: Formal analysis, Writing – review & editing. MS: Formal analysis, Writing – review & editing. WM: Conceptualization, Funding acquisition, Investigation, Methodology, Project administration, Supervision, Validation, Visualization, Writing – review & editing.

## References

[1] Jølving LR Nielsen J Kesmodel US Nielsen RG Beck-Nielsen SS Nørgård BM. Prevalence of maternal chronic diseases during pregnancy—a nationwide population based study from 1989 to 2013. Acta Obstet Gynecol Scand. (2016) 95:1295–304. doi: 10.1111/aogs.13007, PMID: 27560844

[2] Lee SI Azcoaga-Lorenzo A Agrawal U Kennedy JI Fagbamigbe AF Hope H . Epidemiology of pre-existing multimorbidity in pregnant women in the UK in 2018: a population-based cross-sectional study. BMC Pregnancy Childbirth. (2022) 22:120. doi: 10.1186/s12884-022-04442-3, PMID: 35148719 PMC8840793

[3] Brown CC Adams CE George KE Moore JE. Associations between comorbidities and severe maternal morbidity. Obstet Gynecol. (2020) 136:892–901. doi: 10.1097/AOG.0000000000004057, PMID: 33030867 PMC8006182

[4] Heidemann C Scheidt-Nave C Beyer AK Baumert J Thamm R Maier B . (2021). Gesundheitliche Lage von Erwachsenen in Deutschland Ergebnisse zu ausgewählten Indikatoren der Studie GEDA 2019/2020-EHIS. Robert Koch-Institut. Available at: https://edoc.rki.de/handle/176904/8749

[5] Correa-de-Araujo R Yoon SS. Clinical outcomes in high-risk pregnancies due to advanced maternal age. J Women's Health. (2021) 30:160–7. doi: 10.1089/jwh.2020.8860 PMC8020515 33185505

[6] van der Kooi ALLF Mulder RL Hudson MM Kremer LCM Skinner R Constine LS . Counseling and surveillance of obstetrical risks for female childhood, adolescent, and young adult cancer survivors: recommendations from the international late effects of childhood Cancer guideline harmonization group. Am J Obstet Gynecol. (2021) 224:3–15. doi: 10.1016/j.ajog.2020.05.058, PMID: 32502557

[7] Moons P Bovijn L Budts W Belmans A Gewillig M. Temporal trends in survival to adulthood among patients born with congenital heart disease from 1970 to 1992 in Belgium. Circulation. (2010) 122:2264–72. doi: 10.1161/CIRCULATIONAHA.110.946343, PMID: 21098444

[8] Moons P Bratt EL De Backer J Goossens E Hornung T Tutarel O . Transition to adulthood and transfer to adult care of adolescents with congenital heart disease: a global consensus statement of the ESC Association of cardiovascular nursing and allied professions (ACNAP), the ESC working group on adult congenital heart disease (WG ACHD), the Association for European Paediatric and Congenital Cardiology (AEPC), the Pan-African Society of Cardiology (PASCAR), the Asia-Pacific pediatric cardiac society (APPCS), the inter-American Society of Cardiology (IASC), the Cardiac Society of Australia and new Zealand (CSANZ), the International Society for Adult Congenital Heart Disease (ISACHD), the world heart federation (WHF), the European congenital heart disease organisation (ECHDO), and the global Alliance for rheumatic and congenital hearts (global ARCH). Eur Heart J. (2021) 42:4213–23. doi: 10.1093/eurheartj/ehab388, PMID: 34198319 PMC8560210

[9] Mandalenakis Z Giang KW Eriksson P Liden H Synnergren M Wåhlander H . Survival in children with congenital heart disease: have we reached a peak at 97%? JAHA. (2020) 9:e017704. doi: 10.1161/JAHA.120.017704, PMID: 33153356 PMC7763707

[10] Khizroeva J Nalli C Bitsadze V Lojacono A Zatti S Andreoli L . Infertility in women with systemic autoimmune diseases. Best Pract Res Clin Endocrinol Metab. (2019) 33:101369. doi: 10.1016/j.beem.2019.101369, PMID: 31837981

[11] Nair M Knight M Kurinczuk J. Risk factors and newborn outcomes associated with maternal deaths in the UK from 2009 to 2013: a national case–control study. BJOG Int J Obstet Gy. (2016) 123:1654–62. doi: 10.1111/1471-0528.13978, PMID: 26969482 PMC5021205

[12] Admon LK Winkelman TNA Heisler M Dalton VK. Obstetric outcomes and delivery-related health care utilization and costs among pregnant women with multiple chronic conditions. Prev Chronic Dis. (2018) 15:170397. doi: 10.5888/pcd15.170397 PMC5814150 29420168

[13] Nair M Kurinczuk J Brocklehurst P Sellers S Lewis G Knight M. Factors associated with maternal death from direct pregnancy complications: a UK national case–control study. BJOG Int J Obstet Gy. (2015) 122:653–62. doi: 10.1111/1471-0528.13279, PMID: 25573167 PMC4674982

[14] Ferreira EC Costa ML Pacagnella RC Silveira C Andreucci CB Zanardi DMT . Multidimensional assessment of women after severe maternal morbidity: the COMMAG cohort study. BMJ Open. (2020) 10:e041138. doi: 10.1136/bmjopen-2020-041138, PMID: 33303455 PMC7733206

[15] Centers for Disease Control and Prevention (2023). Severe maternal morbidity in the United States. Available at: https://www.cdc.gov/reproductivehealth/maternalinfanthealth/severematernalmorbidity.html#anchor_References (Accessed November 4, 2023).

[16] Chen J Cox S Kuklina EV Ferre C Barfield W Li R. Assessment of incidence and factors associated with severe maternal morbidity after delivery discharge among women in the US. JAMA Netw Open. (2021) 4:e2036148. doi: 10.1001/jamanetworkopen.2020.36148, PMID: 33528553 PMC7856547

[17] Lammers AE Diller GP Lober R Möllers M Schmidt R Radke RM . Maternal and neonatal complications in women with congenital heart disease: a nationwide analysis. Eur Heart J. (2021) 42:4252–60. doi: 10.1093/eurheartj/ehab571, PMID: 34638134

[18] Hui D Hladunewich MA. Chronic kidney disease and pregnancy. Obstet Gynecol. (2019) 133:1182–94. doi: 10.1097/AOG.0000000000003256 31135733

[19] Jethwa H Lam S Smith C Giles I. Does rheumatoid arthritis really improve during pregnancy? A systematic review and meta analysis. J Rheumatol. (2019) 46:245–50. doi: 10.3899/jrheum.180226, PMID: 30385703

[20] Andreoli L García-Fernández A Chiara Gerardi M Tincani A. The course of rheumatic diseases during pregnancy. Isr Med Assoc J. (2019) 21:464–70. PMID: 31507122

[21] Salemi G Callari G Gammino M Battaglieri F Cammarata E Cuccia G . The relapse rate of multiple sclerosis changes during pregnancy: a cohort study. Acta Neurol Scand. (2004) 110:23–6. doi: 10.1111/j.1600-0404.2004.00270.x, PMID: 15180803

[22] Kilpatrick SJ Abreo A Gould J Greene N Main EK. Confirmed severe maternal morbidity is associated with high rate of preterm delivery. Am J Obstet Gynecol. (2016) 215:233.e1–7. doi: 10.1016/j.ajog.2016.02.026, PMID: 26899903

[23] Jakobsson M Tapper AM Palomäki O Ojala K Pallasmaa N Ordén MR . Neonatal outcomes after the obstetric near-miss events uterine rupture, abnormally invasive placenta and emergency peripartum hysterectomy—prospective data from the 2009-2011 Finnish NOSS study. Acta Obstet Gynecol Scand. (2015) 94:1387–94. doi: 10.1111/aogs.12780, PMID: 26399783

[24] Weber-Schoendorfer C Kayser A Tissen-Diabaté T Winterfeld U Eleftheriou G Te Winkel B . Fetotoxic risk of AT1 blockers exceeds that of angiotensin-converting enzyme inhibitors: an observational study. J Hypertens. (2020) 38:133–41. doi: 10.1097/HJH.0000000000002233, PMID: 31568057

[25] Vesco KK Ferrante S Chen Y Rhodes T Black CM Allen-Ramey F. Costs of severe maternal morbidity during pregnancy in US commercially insured and Medicaid populations: an observational study. Matern Child Health J. (2020) 24:30–8. doi: 10.1007/s10995-019-02819-z, PMID: 31655962

[26] Gemeinsamer Bundesausschuss (G-BA) Richtlinien des Gemeinsame Bundesausschuss (G-BA). (2023). Available at: https://www.g-ba.de/richtlinien/

[27] Khazaeipour Z Nikbakht-Nasrabadi A Mohammadi N Salehi-Nejad A Shabany M. The childbearing experience of women with spinal cord injury in Iran: a phenomenological study. Spinal Cord. (2018) 56:1184–93. doi: 10.1038/s41393-018-0162-3, PMID: 29904190

[28] Rodrigues L Alves VLP Sim-Simc MMF Surita FG. Perceptions of women with systemic lupus erythematosus undergoing high-risk prenatal care: a qualitative study. Midwifery. (2020) 87:102715. doi: 10.1016/j.midw.2020.102715, PMID: 32447183

[29] Berg M. Pregnancy and diabetes: how women handle the challenges. J Perinat Educ. (2005) 14:23–32. doi: 10.1624/105812405X57552 17273439 PMC1595250

[30] Lange U Schnepp WZ Sayn-Wittgenstein F. Das subjektive Erleben chronisch kranker Frauen in der Zeit von Schwangerschaft, Geburt und Wochenbett—eine Analyse qualitativer Studien. Z Geburtshilfe Neonatol. (2015) 219:161–9. doi: 10.1055/s-0034-1398632 26039501

[31] Gemeinsamer Bundesauschuss (2023). Richtlinien des Gemeinsamen Bundesausschusses über die ärztliche Betreuung während der Schwangerschaft und nach der Entbindung 2022. Available at: https://www.g-ba.de/richtlinien/19/

[32] Lowe S Nelson-Piercy C Rosene-Montella K. Training in obstetric medicine: a global issue. Obstet Med. (2009) 2:91–2. doi: 10.1258/om.2009.09e002, PMID: 27582820 PMC4989758

[33] Chan AW Tetzlaff JM Altman DG Laupacis A Gøtzsche PC Krleža-Jerić K . SPIRIT 2013 statement: defining standard protocol items for clinical trials. Ann Intern Med. (2013) 158:200–7. doi: 10.7326/0003-4819-158-3-201302050-00583, PMID: 23295957 PMC5114123

[34] Calvert M Kyte D Mercieca-Bebber R Slade A Chan AW King MT . Guidelines for inclusion of patient-reported outcomes in clinical trial protocols: the SPIRIT-PRO extension. JAMA. (2018) 319:483. doi: 10.1001/jama.2017.21903 29411037

[35] Calvert M King M Mercieca-Bebber R Aiyegbusi O Kyte D Slade A . SPIRIT-PRO extension explanation and elaboration: guidelines for inclusion of patient-reported outcomes in protocols of clinical trials. BMJ Open. (2021) 11:e045105. doi: 10.1136/bmjopen-2020-045105, PMID: 34193486 PMC8246371

[36] von Elm E Altman DG Egger M Pocock SJ Gøtzsche PC Vandenbroucke JP. The strengthening the reporting of observational studies in epidemiology (STROBE) statement: guidelines for reporting observational studies. Lancet. (2007) 370:1453–7. doi: 10.1016/S0140-6736(07)61602-X 18064739

[37] Vandenbroucke JP von Elm E Altman DG Gøtzsche PC Mulrow CD Pocock SJ . Strengthening the reporting of observational studies in epidemiology (STROBE): explanation and elaboration. PLoS Med. (2007) 4:e297. doi: 10.1371/journal.pmed.0040297, PMID: 17941715 PMC2020496

[38] Ludwig K Graf von der Schulenburg JM Greiner W. German value set for the EQ-5D-5L. PharmacoEconomics. (2018) 36:663–74. doi: 10.1007/s40273-018-0615-8, PMID: 29460066 PMC5954069

[39] Hollins Martin CJ Martin CR. Development and psychometric properties of the birth satisfaction scale-revised (BSS-R). Midwifery. (2014) 30:610–9. doi: 10.1016/j.midw.2013.10.006, PMID: 24252712

[40] Hartmann C Rose M Weichert A Weißhaupt K. The ICHOM standard set for pregnancy and childbirth—translation and linguistic adaptation for Germany. Geburtshilfe Frauenheilkd. (2022) 82:747–54. doi: 10.1055/a-1666-0429, PMID: 35815101 PMC9262632

[41] Lachin JM. Sample size evaluation for a multiply matched case–control study using the score test from a conditional logistic (discrete Cox PH) regression model. Stat Med. (2008) 27:2509–23. doi: 10.1002/sim.3057 17886235 PMC3626499

[42] EuroQol Research Foundation EQ-5D-5L user guide 2019. (2023) Available at: https://euroqol.org/publications/user-guides

[43] Guglielminotti J Landau R Wong CA Li G. Patient-, hospital-, and neighborhood-level factors associated with severe maternal morbidity during childbirth: a cross-sectional study in New York state 2013–2014. Matern Child Health J. (2019) 23:82–91. doi: 10.1007/s10995-018-2596-9, PMID: 30014373

[44] Callaghan WM Creanga AA Kuklina EV. Severe maternal morbidity among delivery and postpartum hospitalizations in the United States. Obstet Gynecol. (2012) 120:1029–36. doi: 10.1097/AOG.0b013e31826d60c5 23090519

[45] Blümel M Spranger A Achstetter K Maresso A Busse R. Germany: health system review. Health Syst Transit. (2020) 22:1–272. PMID: 34232120

[46] Iezzoni LI Wint AJ Smeltzer SC Ecker JL. Recommendations about pregnancy from women with mobility disability to their peers. Womens Health Issues. (2017) 27:75–82. doi: 10.1016/j.whi.2016.09.004, PMID: 27773531 PMC5177507

[47] Hammarberg K Stocker R Romero L Fisher J. Pregnancy planning health information and service needs of women with chronic non-communicable conditions: a systematic review and narrative synthesis. BMC Pregnancy Childbirth. (2022) 22:236. doi: 10.1186/s12884-022-04498-1, PMID: 35317730 PMC8941766

[48] EuroQol Group. EuroQol—a new facility for the measurement of health-related quality of life. Health Policy. (1990) 16:199–208. doi: 10.1016/0168-8510(90)90421-9, PMID: 10109801

[49] Rabin R de Charro F. EQ-SD: a measure of health status from the EuroQol group. Ann Med. (2001) 33:337–43. doi: 10.3109/07853890109002087 11491192

[50] Huizink AC Delforterie MJ Scheinin NM Tolvanen M Karlsson L Karlsson H. Adaption of pregnancy anxiety questionnaire–revised for all pregnant women regardless of parity: PRAQ-R2. Arch Womens Ment Health. (2016) 19:125–32. doi: 10.1007/s00737-015-0531-2, PMID: 25971851 PMC4728175

[51] Chen S Zhao S Dalman C Karlsson H Gardner R. Association of maternal diabetes with neurodevelopmental disorders: autism spectrum disorders, attention-deficit/hyperactivity disorder and intellectual disability. Int J Epidemiol. (2021) 50:459–74. doi: 10.1093/ije/dyaa212, PMID: 33221916 PMC8128461

[52] Declercq ER Cabral HJ Cui X Liu CL Amutah-Onukagha N Larson E . Using longitudinally linked data to measure severe maternal morbidity. Obstet Gynecol. (2022) 139:165–71. doi: 10.1097/AOG.0000000000004641, PMID: 34991121 PMC8820447

