# Supplementary material for: Obstetric Medicine: the protocol for a prospective three-dimensional cohort study to assess maternity care for women with pre-existing conditions (ForMaT)
Source: Front Med (Lausanne). 2024 Jan 11;10:1258716. doi: 10.3389/fmed.2023.1258716 (PMC10808351; doi:10.3389/fmed.2023.1258716)
Supplement: Supplementary file 1 [file Data_Sheet_1.docx]

*Supplementary Material*

Table 1: List of pre-existing conditions included in study group S, adapted from Lee et al. (1)

| Pre-existing conditions included in the study group S | |
| --- | --- |
| Cardiovascular | Congenital malformations of the circulatory system |
|  | Atrioventricular block (second and third degree) |
|  | Ventricular flutter and fibrillation |
|  | Sick sinus syndrome |
|  | Pre-existing hypertension (on medication before conception) |
|  | Presence of cardiac and vascular implant and graft |
|  | Cardiac arrest with successful resuscitation |
|  | Cardiac sarcoidosis |
|  | Marfan syndrome |
|  | Long QT syndrome |
|  | Wolff-Parkinson-White syndrome |
|  | Heart failure |
|  | Cardiomyopathy |
| Rheumatology | Systemic lupus erythematosus |
|  | Vasculitis |
|  | Rheumatoid arthritis, Spondylarthritis, Psoriatic arthritis |
|  | Systemic sclerosis |
|  | Sjögren syndrome |
|  | Ehlers-Danlos-syndrome |
| Neurology | Multiple sclerosis |
|  | Myasthenia gravis |
|  | Muscular dystrophy |
|  | Sequelae of cerebrovascular disease |
|  | Subarachnoid hemorrhage |
|  | Intracerebral hemorrhage |
|  | Cerebral infarction |
|  | Congenital brain malformations |
|  | Cerebrovascular diseases |
|  | Cerebral aneurysm and dissections |
|  | Somatoform disorder (on medication, e.g., fibromyalgia, chronic pain syndrome, chronic fatigue syndrome) |
|  | Idiopathic intracranial hypertension (symptomatic within 6 months before conception or on treatment) |
|  | Epilepsy |
|  | Presence of cerebrospinal fluid drainage device |
|  | Spina bifida |
| Psychiatry | Anxiety disorder, panic disorder (on ≥2 medication) |
|  | Depression (on ≥2 medication) |
|  | Schizophrenia |
|  | Mental and behavioral disorders due to substance use |
|  | Psychosis, psychotic disorders |
|  | Bipolar disorder |
|  | Other mental health conditions (on ≥2 medication) |
| Oncology | All malignant tumors |
|  | History of malignant tumor with systemic or radiation treatment |
| Gastroenterology | Portal hypertension |
|  | Fibrosis and cirrhosis of liver |
|  | Primary biliary cholangitis |
|  | Symptomatic cholelithiasis |
|  | Hepatitis (autoimmune, viral, chronic, alcoholic) |
|  | Sequelae of viral hepatitis |
|  | Inflammatory bowel disease (on medication) |
|  | Peptic ulcer disease |
|  | History of bariatric surgery |
| Hematology | Thrombocytopenia (e.g., immune thrombocytopenia, thrombotic thrombocytopenic purpura) |
|  | Thrombophilia (e.g., homozygous factor V Leiden mutation, Antithrombin-III deficiency) |
|  | Hemolytic anemia (e.g., thalassemia, sickle cell disease) |
|  | Von Willebrand disease |
|  | Hereditary deficiency of clotting factors, including hemophilia |
|  | History of pulmonary embolism |
|  | History of venous thromboembolism |
|  | Venous thromboembolism |
|  | Pulmonary embolism |
| Endocrinology | Congenital metabolic disorders (e.g., phenylketonuria) |
|  | Pre-existing diabetes mellitus (type 1, type 2, MODY) |
|  | Hyperthyroidism (on medication <6 months before conception) |
|  | Hypo- and Hyperparathyroidism |
|  | Pituitary disorders |
|  | Adrenal benign tumors |
| Pulmonology | COPD (on medication) |
|  | Sleep apnea (on treatment) |
|  | Pulmonary sarcoidosis (if treatment <6 months before conception) |
|  | Bronchial asthma (symptomatic and on ≥2 medication within 6 months before conception) |
|  | Pulmonary fibrosis, interstitial lung disease |
|  | Cystic fibrosis |
|  | Tuberculosis (with pulmonary functional impairment) |
| Ophthalmology | Blindness |
|  | Retinal detachment |
| Orthopedics | Osteoporosis with fractures |
|  | Scoliosis with postsurgical status |
| Nephrology/Urology | Chronic kidney disease |
|  | Glomerulonephritis |
|  | Hereditary kidney diseases (e.g., autosomal dominant polycystic kidney disease, Alport syndrome, Fabry disease) |
|  | Nephrotic syndrome |
|  | Congenital anomalies of the kidney and urinary tract (CAKUT) |
| Other | Human immunodeficiency viral (HIV) infection |
|  | Turner syndrome |
|  | Postsurgical state after correction of relevant congenital malformation (e.g., gastroschisis, imperforate anus) |
|  | Postsurgical state after transplantation |

Table 2: List of pre-existing conditions excluded from recruitment in study group S

| Pre-existing conditions not included in the study (if occurring exclusively) | |
| --- | --- |
| Neurology | Migraine |
|  | Chronic headache (e.g., cluster or tension headache) |
| Psychiatry | Anxiety/Panic disorder (with ≤1 medication, includes phobia, post-traumatic stress disorder) |
|  | Depression (with ≤1 medication) |
|  | Eating disorder |
|  | Neurodevelopmental disorder (without medication, e.g., Autism, Learning difficulties, attention deficit hyperactivity disorder) |
| Dermatology | Atopic eczema |
|  | Psoriasis vulgaris of the skin |
|  | Autoimmune skin disease (vitiligo, alopecia areata) |
|  | Other (seborrheic dermatitis, rosacea, hidradenitis suppurativa, lichen planus) |
| Ear, Nose, Throat | Allergic rhino conjunctivitis |
| Gynecology | Polycystic ovarian syndrome |
|  | Endometriosis |
|  | Leiomyoma (uterine fibroids) |
| Orthopedics | Scoliosis without surgical treatment |
|  | Chronic back pain |
| Cardiovascular | Essential hypertension (without medication before onset of pregnancy) |
|  | Atrioventricular block, first degree |
| Hemostaseology | Factor V deficiency, heterozygous |
|  | Prothrombin gene mutation, heterozygous |
|  | Elevated Lipoprotein(a) |
|  | PAI-1 gene polymorphism |
| Endocrinology | Substituted hypothyroidism |
| Pulmonology | Asthma (uncomplicated with ≤1 medication and stable disease ≥6 months before conception) |
|  | History of tuberculosis without pulmonary impairment |
|  | Sleep apnea without treatment |
|  | Sarcoidosis (without treatment and stable disease ≥ 6 months before conception) |
|  | COPD (without therapy) |

Table 3: Obstetric risk factors as inclusion criteria for group K1 (at least one of the following criteria must be present)

| Obstetric risk factors in group K1 | |
| --- | --- |
| Hypertensive disorders of pregnancy | Pregnancy-induced hypertension |
|  | Preeclampsia/HELLP syndrome |
|  | Eclampsia during pregnancy |
| Gestational diabetes | Dietary-dependent |
|  | Insulin-dependent |
| Placenta/vasa previa |  |
| Suspected PAS |  |
| Twin pregnancy |  |
| Fetal malpresentation |  |
| Threatened preterm delivery, including | Preterm labor (GA <37+0) |
|  | Preterm premature rupture of membranes (PPROM, GA 22+0 to 32+0) |
|  | Cervical insufficiency (GA 22+0 to 32+0 and cervical length below 5th percentile) |
| Idiopathic cholestasis of pregnancy |  |
| Pregnancy induced thrombocytopenia |  |
| History of one of the following conditions: | Cesarean section |
|  | Transmural uterine surgery (e.g., myomectomy) |
|  | OASIS |
|  | IUFD |
|  | Preeclampsia/HELLP syndrome/eclampsia |
|  | PPH (requiring blood transfusion) |
|  | Shoulder dystocia (a minimum of one maneuver has been performed) |
|  | PAS |

Abbreviations:

COPD: Chronic obstructive pulmonary disease; FVL: Factor V Leiden Mutation; GA: Gestational age; HELLP: Hemolysis, Elevated Liver enzymes, Low Platelet count; IUFD: Intrauterine fetal demise; K1: Name of additional group; MODY: Maturity-onset diabetes of the young; OASIS: Obstetrical anal sphincter injuries; PPH: Postpartum hemorrhage; PPROM: Preterm premature rupture of membranes; S: Name of study group

References:

1. Lee SI, Azcoaga-Lorenzo A, Agrawal U, Kennedy JI, Fagbamigbe AF, Hope H, et al. Epidemiology of pre-existing multimorbidity in pregnant women in the UK in 2018: a population-based cross-sectional study. BMC Pregnancy Childbirth. 2022 Dec;22(1):120.
